# A novel diagnostic approach to Paratuberculosis in dairy cattle using near-infrared spectroscopy and aquaphotomics

**DOI:** 10.3389/fcimb.2024.1374560

**Published:** 2024-05-30

**Authors:** Saba Behdad, Abbas Pakdel, Reza Massudi

**Affiliations:** ^1^ Department of Animal Science, College of Agriculture, Science and Research Branch, Islamic Azad University, Tehran, Iran; ^2^ Department of Animal Science, College of Agriculture, Isfahan University of Technology, Isfahan, Iran; ^3^ Laser and Plasma Institute, Shahid Beheshti University, Tehran, Iran

**Keywords:** Paratuberculosis, Johne’s disease, near-infrared spectroscopy, aquaphotomics, blood plasma, dairy cattle

## Abstract

**Introduction:**

As a contagious and chronic disease in the livestock industry, Paratuberculosis is a significant threat to dairy herds’ genetic and economic resources. Due to intensive breeding and high production of dairy cattle, the incidence and prevalence are higher. Developing non-destructive diagnostic methods for the early detection and identification of healthy animals is paramount for breeding programs. Conventional methods are almost entirely destructive, have low accuracy, lack precision, and are time-consuming. Near-infrared spectroscopy (NIRS) and aquaphotomics can detect changes in biofluids and thus have the potential to diagnose disease. This study aimed to investigate the diagnostic ability of NIRS and aquaphotomics for Paratuberculosis in dairy cattle.

**Methods:**

Blood plasma from dairy cattle was collected in the NIR range (1,300 nm to 1,600 nm) 60 days before and 100 days to 200 days after calving in two groups, positive and negative, using the same consecutive enzyme-linked immunosorbent assay test results three times as a reference test.

**Results:**

NIRS and aquaphotomics methods invite 100% accuracy, sensitivity, and specificity to detect Paratuberculosis using data mining by unsupervised method, Principal Component Analysis, and supervised methods: Soft Independent Modeling of Class Analogiest, Linear Discriminant Analysis, Quadratic Discriminant Analysis, Partial Least Square–Discriminant Analysis, and Support Vector Machine models.

**Discussion:**

The current study found that monitoring blood plasma with NIR spectra provides an opportunity to analyze antibody levels indirectly via changes in water spectral patterns caused by complex physiological changes, such as the amount of antibodies related to Paratuberculosis by aquagram.

## 1 Introduction

*Mycobacterium avium subspecies Paratuberculosis* (MAP) causes Paratuberculosis or Johne’s disease (JD), a chronic, progressive intestinal disease. Ruminants are infected with a weakly Gram-positive acid-fast bacterium that can spread to the entire herd via horizontal transmission, the fecal-oral route, mainly via fecal contamination of the udder or pasture, water, food, colostrum, and aerosol formation, or vertical transmission via MAP from the infected dam to the embryo by placenta (Lee et al., 2023).

Because MAP has a long incubation period and is transmissible, JD is critical from a socio-economic and public health standpoint (OIE, 2012). It is classified into four stages: silent, subclinical, clinical, and advanced. It can affect calves from the embryonic period to the first months of birth, but clinical symptoms may not appear for years. Other animals are exposed to contamination from infected animals’ feces, the environment, food, and milk during this time. This issue will accelerate the spread of the disease in the herd so that, for every animal in the final stage of the disease, there are one to two in the clinical stage, six to eight in the subclinical stage, and 15 to 25 in the initial stage of the disease (Whitlock and R, 2010). This point highlights the importance of paying particular attention to the Trojan named Johne’s disease to preserve genetic resources.

Because of the more intensive breeding of dairy cattle, the prevalence and incidence of JD are higher in dairy cattle than in beef cattle. Moreover, most beef cattle are slaughtered before they become symptomatic from this disease (Rindi and Garzelli, 2014). JD is a factor that can reduce productive traits like milk and meat production. Also, the fertility rate, one of the functional traits, significantly impacts the herd’s profitability (Arnott et al., 2015).

Paratuberculosis has numerous direct and indirect economic costs. Direct economic loss effects include both visible and invisible consequences. The visible consequences of Paratuberculosis infection include reduced growth rate, lower meat and milk production, premature culling of dairy cows, higher mortality, and increased costs due to compensation. The invisible effects include reduced fertility or infertility, disease control costs, diagnostic test costs, abortions, infected calves born, susceptibility to other diseases, and veterinary costs. The indirect economic impacts of this disease include the cost of disease control, revenue foregone due to restricted market access, export losses, losses to other sectors in the supply chain and consumers, impact on animals’ health and welfare, marketing, and public health–related issues, productivity reduction, loss of business and market, decrease in market value, and food insecurity (Miglior et al., 2017; Barratt et al., 2019). The first step in controlling the spread of infection will be rapid identification of the infected animals, separation from the herd, and vaccination of herd members. Additionally, infected animals entering the field will be a significant risk factor in spreading bacteria (Chaubey et al., 2016).

It is critical to use the proper tests and diagnostic approaches for MAP infection and spread in the shortest amount of time (McGregor, 2015). The diagnostic tests for MAP infection are primarily infection detection tests and tests to identify the host’s immune response to the bacterium. Two techniques of bacterial culture and tracking its molecular component by polymerase chain reaction (PCR) technique are used to diagnose MAP strains (Assessment of surveillance and control of Johne’s disease in farm animals in GB, 2002). The molecular techniques of enzyme-linked immunosorbent assay (ELISA), complement fixation test, and agar gel immunodiffusion have been frequently used to assess the host immune response (Sardaro et al., 2017). Still, the level of accuracy and sensitivity of each of these techniques is very different. It is sometimes recommended to use a set of tests, especially in more advanced stages of the disease. However, it is worth noting that costs and logistics (Rieger et al., 2021) can influence the choice of tests. Of course, it should be noted that the mentioned molecular tests are very effective in early screening and tracking and that histopathological studies will allow for a definitive and accurate diagnosis of the disease, particularly its tissue effects. According to the studies, whereas the ELISA test for the detection of antibodies directed against MAP is the most cost-effective tool, PCR or fecal culture (FC) testing is preferred for lowering prevalence, and both are assumed to be more sensitive for low-shedding animals (Robins et al., 2015; Smith et al., 2017).

Others state that combining ELISA and PCR and serially interpreting them would be the most cost-effective (Aly et al., 2012). As a result, the lack of early and accurate diagnostic tests and MAP’s inherent resistance to antibiotics and disinfectants has made infection control extremely difficult, turning JD into a global challenge. Thus, developing an accurate diagnostic method for distinguishing healthy animals from infected ones based on the disease agent the antibody produced and being able to show the animal’s state at each stage of the disease is a global necessity.

Nowadays, using modern physical methods in disease diagnosis is more preferred and desirable due to their accuracy, non-invasiveness, cost and time effectiveness, and fewer side effects than chemical methods. Recent research studies to diagnose JD based on modern physical methods include fluorescence imaging (Le Puil et al., 2006); near-infrared spectroscopy (NIRS) (Norby et al., 2006); flow cytometry (Allen et al., 2011); Raman spectroscopy (Yakes et al., 2008); transabdominal ultrasonography (Tooloei et al., 2016); hyperspectral image analysis and NIRS (Smith, 2016); confocal microscope (Mathie, 2017); monitoring the change of gene expression pattern in salivary glands (Sanjay Mallikarjunappa et al., 2019); UV-VIS spectroscopy and gold nanoparticle (Agrawal et al., 2020); and immunohistochemistry, immunofluorescence, and immunomagnetic separation (Karuppusamy et al., 2021). Despite these studies, developing a non-invasive, accurate, fast, available, and cost-effective gold method for diagnosing JD and its four stages, particularly in its early stages, remains a global challenge.

NIRS, as one of the physical and non-invasive methods, without sample preparation or chemical pollution, with high accuracy and based on molecular bond vibration measuring, has been widely used in the last 50 years in agriculture to quantify nutrient composition for crops and in quality control across the food industry and pharmaceutical products by establishing the interaction of electromagnetic waves with biological materials. Water is cited as one of the disadvantages of NIRS in aqua systems because it could alter sample spectra, hide absorbance bonds, and shift absorbance bonds. Another is the inability to detect the amount smaller than 500 ppm in solution. However, in the previous decade, the application of this method has grown significantly to include structural analysis of water. The aquaphotomics approach to analyzing water and aqueous systems like plasma, serum, urine, and milk spectra provides a unique opportunity to describe the complex state of water using its multidimensional NIR spectra (Munćan et al., 2019).

Aquaphotomics as a novel scientific discipline founded by Professor Roumiana Tsenkova in 2005, involving the study of water and aqueous system, using light–water interaction to extract information about the structure of water, is composed of many different waters molecular information by using water absorbance bands, which found to be huge source information on the subject of the structural and related function properties of aqueous system. It is a complementary “omics” discipline with the large-scale, comprehensive studies about water as “collective matter and energy mirror” of the rest of the aqueous system that has memory and conciseness to detect perturbation and change the arrangement to keep the stability of biosystem. Whereas genomics studies genes, proteomics studies proteins, glycomics studies carbohydrates, and lipidomics studies lipids, aquaphotomics explores the roles, relationships, and functions of the water—an equally important biomolecule and one of nature’s fundamental building blocks. Water, a natural matrix of any aqueous or biological system, changes its absorbance pattern every time it adapts to a physical or chemical change in the system itself or its environment. Small quantities or structural change of other molecules present in the aqueous system, so the information is to extract about not only water structure but also other components present in water or the state of the system as a whole. Aquaphotomics changes the treat of water in spectroscopy to opportunity to achieve much valuable information, and, in this role, water acts like a sensor and amplifier, especially for low concentration amounts, and detects them and their changes (Tsenkova et al., 2018; Muncan and Tsenkova, 2019).

Aquaphotomics utilizes the high sensitivity of water hydrogen bonds; accordingly, every aqueous system is a dynamic arrangement of water molecule network hydrogen-bonded to other constituents and influenced by perturbation like infection. Any internal or external perturbation of the aqueous system results in changes of water molecular conformations, which, in turn, produce changes in the corresponding NIR spectra at their respective water absorbance bands. In the range of 1,300 nm to 1,600 nm, 12 water absorbance bands more important. Water spectral pattern as a holistic biomarker, which relates certain water structures with functionalities of the respective biological system, thus opens new directions to ward non-destructive quality monitoring applications and non-invasive biodiagnosis (Tsenkova et al., 2018; Muncan and Tsenkova, 2019).

Light–water interaction spectroscopic methods produce complex multidimensional spectral data, which require data processing and analysis to extract hidden information from water spectra in aquaphotomics. Preprocessing and chemometrics methods remove unwanted influences and extract water absorbance spectral pattern related to the perturbation of interest by identification of activated water absorbance bands. NIRS, non-destructive tool, offers the advantages of *in vivo* spectral monitoring of living objects. Aquaphotomics combined with time-resoled NIRS allows a better understanding of biological functions and underlying water dynamics (Tsenkova et al., 2018).

NIRS and aquaphotomics models need to chemical methods as a reference test for calibration and then can predict the samples by models. In this study, ELISA test selects for reference test, and, for increasing of accurate, samples select according to the three consequent results of tests. Applying two chemometrics methods for the evaluation of one aspect of the experimental study demonstrates stability of the applied methodology, namely, consistency in results (Tsenkova et al., 2018). In this way, we apply preprocessing and five methods: Principal Component Analysis (PCA) as unsupervised method in the first, and, then, Soft Independent Modeling of Class Analogies (SIMCA), Linear Discriminat Analysis (LDA), Quadratic Discriminant Analysis (QDA), Partial Least Square‐ Discriminant Analysis (PLS-DA), and Support Vector Machine (SVM) as supervised methods, for discrimination between healthy and infected dairy cattle by Paratuberculosis, consistent in our results in the total range of 1,300 nm to 1,600 nm and then in 12 water absorbance bands.

Aquaphotomics was applied for discrimination of healthy and mastitic animals based on the spectra of urine, blood, and milk of dairy cows (Tsenkova and Atanassova, 2001; Tsenkova, 2007; Meilina et al., 2009; Morita et al., 2013), estrus detection in cow and panda (Kinoshita et al., 2012a, 2015; Iweka et al., 2020), discrimination of different bacteria strains (Remagni et al., 2013; Slavchev et al., 2015, 2017; Kovacs et al., 2019), pneumonia in dairy calves (Santos-Rivera et al., 2021), and detection of type2 diabetic (Li et al., 2020).

NIRS and aquaphotomics combined with chemometrics based multivariate analysis (MVA) may be able to identify and discriminate the biochemical profile of blood plasma associated with Paratuberculosis infection and to monitor the water molecules of blood plasma response to perturbation of this infection in dairy cattle. According to the function of collective mirror, water, all of the changes in biomolecules or MAP in JD that today measure separately may be monitored by water absorbance bands accurately.

The objectives of this study were to use blood plasma for diagnosis of Paratuberculosis by NIRS and supervised methods and then to use water bonds of blood plasma for biomonitoring and discovering water spectral changes in blood plasma associated with diagnosing Paratuberculosis using Aquaphotomics and chemometrics. The current diagnostic methods for Paratuberculosis require detecting MAP through FC, PCR test, or measuring an adequate level of antibodies through ELISA test, but not accurate in low concentration. Aquaphotomics can detect the low concentration of antibody or other changes in biomolecules by infection of Paratuberculosis perturbation.

Our long-term goal is to create a diagnostic strategy for the detection of dairy cattle in the four stages of JD and then in early detection of Paratuberculosis by this non-invasive, accurate, and fast diagnostic method *in vivo*, thereby contributing to the sustainability of the genetic resources and the global health.

## 2 Methods

### 2.1 Animals and farms

Holstein dairy cows in the second and third lactation periods were used in this study. It should be noted that the ELISA test was not accrued in the early calf due to the special conditions of JD, so the population of that range of cattle is larger than others. Therefore, finding positive cattle is easier. The selected cows are considered free of another disease. The experiment was carried out over one summer. Two physiologic periods in dairy cattle were chosen, one about 60 days before drying from one farm and the other 100 days to 200 days after calving from another in Iran’s Isfahan province.

The cattle to be dried 3 weeks later were chosen randomly among the cows in the second and third lactation periods on the first farm (n = 50). In their second or third lactation periods and about 100 days to 200 days after calving, dairy cows were chosen for the second herd (n = 50). Dairy cows with similar ELISA testing results in three consecutive weeks were chosen on the basis of the history of ELISA testing in the first year. Cows with ELISA test index of less than 20 and greater than 100 were classified as negative and positive, respectively (**Table 1**).

**Table 1:** The characteristics of negative and positive animal groups of Paratuberculosis by ELISA test as a reference test.

Contents	Herd	Total number of cows	Number of cows according to the blood plasma ELISA test	
			Positive	Negative
60 days before drying	1	11	2	9
100 days to 200 days after calving (first week)	2	11	8	3
100 days to 200 days after calving (second week)	2	11	8	3
100 days to 200 days after calving (third week)	2	11	8	3
Total	2	22	10	12

### 2.2 Blood collection

This research was approved by the SRBIAU–Institution Animal Science (SRB-11-3997) and complied with the institutional, national, and international Animal Research: Reporting In Vivo Experiments guidelines (ARRIVE). The animals studied in this research were not separated from the herd. The farms had a routine program for monitoring JD, and the blood samples taken by staff for ELISA tests were also used for spectroscopy. Four blood samples were collected for each cow in the first farm (n = 200) 3 weeks before drying and 1 week after calving, and 11 animals were selected. In the second farm, blood sample tubes were collected for cows whose ELISA tests were confirmed (n = 150), and, then, blood samples were taken three times within 3 weeks from selected cows (n = 33). Blood samples were collected via caudal venipuncture into two commercial blood collection tubes containing the anticoagulant Ethylenediaminetetraacetic acid and immediately placed on ice (n = 383). Two tubes were centrifuged at 4,000 rpm for 20 min to separate the plasma, and the plasma was stored in six 2-mL microtubes at −23°C until NIRS analysis. The second tube was used for the ELISA test with an IDEXX kit.

### 2.3 Reference analysis (ELISA test)

Models were created and evaluated for the selected samples by comparing the estimated and reference values in three results of consecutive blood plasma. ELISA tests during the 3 weeks were considered for the reference analysis.

The IDXX kit, as an ELISA kit, has enzyme immunoassay for the detection of antibodies directed against MAP in bovine individual serum, plasma, and milk samples. First, coated plates were obtained and the sample position was recorded. The negative control (NC) was diluted 1:20 in the dilution buffer N.12 and was dispensed in one well. The positive control (PC) was diluted 1:20 in the dilution buffer N.12 and was dispensed in two wells. Plasma samples were diluted 1:20 in the dilution buffer N.12 and, after, contents used a microplate shaker were homogenized and incubated 15 min to 2 h at 18°C to 26°C; 100 µL from each well transferred to the preplate to appropriate wells of the coated microplate. After, contents of the wells by microplate shaker were homogenized, covered, and incubated for 45 min at 18°C to 26°C. Then, the solution was removed, and each well was washed with approximately 300 µL of wash solution three to five times. After that, 100 µL of conjugate was dispensed into each well, covered, and incubated for 30 min. The solution was removed, and each well was washed with approximately 300 µL of wash solution three to five times. Then, 100 µL of TMB substrate N.9 was dispensed into each well and incubated 10 min 18°C to 26°C away from direct light; 100 µL of stop solution N.3 was dispensed into each well. Optical densities values of samples and controls at 450 nm were measured and recorded. For the calculation of controls, Equation 1 was applied, and, for plasma samples, Equation 2 was applied (IDXX Paratuberculosis screening 06-07130-27 manual):


(1) $$\begin{array}{c} \text{Control: }\text{PC}\bar{\text{X}}\;=\frac{\;\text{PC}1\left(450\right)+\text{PC}2\left(450\right)}{2}\\ Validity\;criteria\text{:}\frac{\text{PC}\bar{\text{X}}}{\text{NC}}\;\text{A}\left(450\right)\geq 3.00\text{ and }PC\bar{X}\geq 0.350 \end{array}$$

(2) $$\begin{array}{c} Interpretation\;for\;plasma\;samples:\\ \text{Sample / Control}\text{(S/P)\%}=100*\frac{\text{sample A}\left(450\right)-\text{NC A}\left(450\right)}{\text{PC}\bar{\text{X}}-\text{NC A}\left(450\right)}\\ \text{Negative}:S/P\%<45\%\\ \text{Suspect}:45\%<S/P\%<55\%\\ \text{Positive: }S/P\%\geq 55\% \end{array}$$

In this study, to increase the accurate of reference test, the animals had the same results of S/P in three consequent test  ≥100 and  ≤20 , considered as positive and negative, respectively.

### 2.4 NIR spectral signature collection

Blood plasma NIR absorbance spectra were collected using a spectrophotometer (UV-VIS-NIR 3600, Shimadzu CO. Japan) equipped with a quartz cuvette having a 1-mm optical path length (n = 79). The samples were thawed over ice for 15 min and warmed between hands for approximately 1 min before NIR spectra collection. NIR spectrum acquired in the range of 1,280 nm to 1,630 nm (interval = 0.5 nm; single scan; very slow). Before collecting plasma spectra, a reference spectrum was captured from two empty cuvettes, followed by one empty cuvette and one containing distilled water. Three independent spectral signatures were collected per sample, with the cuvette being repacked with plasma between each replicate.

### 2.5 Multivariate analysis

The chemometrics-based multivariate analysis (MVA) was performed on the first overtone region of the near-infrared spectrum in the vibrational combination band between 1,300 nm and 1,600 nm using Unscrambler X v.10.5. The mathematical pretreatments of the linear baseline correction; standard normal variate (SNV) with detrending polynomial order and a first derivative (symmetric Savitzky–Golay smoothing, points = 12); smoothing, normalized, multiplicative scatter correction (MSC); and spectroscopic (absorbance to transmittance) are applied to all the databases described next.

A balanced dataset (n = 301) was created by spectral signatures for each category (healthy, or negative, infected, or positive) (**Table 2**). This dataset contains spectra from all 22 cattle and was used to perform PCA, SIMCA, discriminant analysis: LDA, QDA, PLS-DA, and SVM, followed by aquaphotomics analyses. In the supervised analysis, datasets were created by positive and negative groups to test mathematical preprocessing and modeling bias against the null hypothesis (no biological signature can differentiate between samples from two classes).

**Table 2:** The results of supervised methods (SIMCA, LDA-PCA, QDA-PCA, PLS-DA, and SVM) to detect the positive and negative groups of Paratuberculosis in the range of 1,300 nm to 1,600 nm and 12 water absorbance bands (WAMACs) according the results of ELISA test as a reference test.

Model	Range	Contents	Predicted model		
			Full data: full validation	Calibration: full validation	Test: cross-validation
SIMCA	1,300–1,600 nm	Negative			100 (7/7)
		Positive			91 (10/11)
		Total accuracy			94.5
		Pretreatment			Normalize
	12 Water absorbance bands	Negative			100
		Positive			91
		Total accuracy			94.5
		Pretreatment			Smoothing
LDA	1,300–1,600 nm	Negative	87.5 (28/32)	88 (22/25)	100 (7/7)
		Positive	91 (41/45)	88.2 (30/34)	100 (11/11)
		Total accuracy	89.5	88.1	100
		PC	20	20	10
		Pretreatment	–	–	–
	12 Water absorbance bands	Negative	97 (31/32)	96 (24/25)	100 (7/7)
		Positive	91 (41/45)	88.2 (30/34)	100 (11/11)
		Total accuracy	93.5	91.5	100
		PC	20	20	20
		Pretreatment	–	–	–
QDA	1,300–1,600 nm	Negative	100 (32/32)	100 (25/25)	100 (7/7)
		Positive	98 (44/45)	97 (33/34)	100 (11/11)
		Total accuracy	98.8	98.3	100
		Pretreatment	–	–	–
		PC	20	20	20
	12 Water absorbance bands	Negative	100 (32/32)	100 (25/25)	100 (7/7)
		Positive	98 (44/45)	97 (33/34)	100 (11/11)
		Total accuracy	98.8	98.3	100
		Pretreatment	MSC	MSC	MSC
		PC	18	20	20
PLS-DA	1,300–1,600 nm	Negative	100 (32/32)	100 (25/25)	100 (7/7)
		Positive	98 (44/45)	97 (33/34)	100 (11/11)
		Total accuracy	98.8	98.3	100
		Pretreatment	Normalized	Normalized	Normalized
		R^2^ - RSME	78% - 0.02	84% - 0.02	81% - 0.02
	12 Water absorbance bands	Negative	94 (30/32)	96 (24/25)	100 (7/7)
		Positive	91 (41/45)	94 (32/34)	82 (9/11)
		Total accuracy	92%	95%	89%
		Pretreatment	Baseline	Baseline	Baseline – MSC - SNV
		R^2^ - RSME	70% - 0.04	71.6% - 0.28	66% - 0.29
SVM	1,300–1,600 nm	Negative	97 (31/32)	96 (24/25)	100
		Positive	100 (45/45)	100 (34/34)	100
		Total accuracy	99	98 (58/59)	100
		Pretreatment	1st derivation	1st derivation	1st derivation
		R^2^ - RMSC	97% - 0.1	96% - 0.1	
	12 Water absorbance bands	Negative	100 (32/32)	100 (25/25)	100
		Positive	98 (44/45)	97 (33/34)	100
		Total accuracy	98.8	98 (58/59)	100
		Pretreatment	Normalized	Normalized	Normalized
		R^2^ - RMSC	95% - 0.1	96%-0.1	

The samples were randomly divided into two subsets: a calibration subset for the internal validation set (75%) and a test subset for the external validation set (25%). This calibration equation was derived from various sample sets: each sample set includes a calibration set of positive (≥ 100) and a calibration set of negative samples (≤ 20).

### 2.6 Principal component analysis

PCA is an unsupervised MVA and a well-known statistical method for reducing the dimensionality of datasets, explaining variation in data by ignoring the data label, assisting in pattern detecting in spectral behavior, and finding excluded data (Ghasemi et al., 2013). The PCA was applied to the dataset, and the calibration sets were created for the discriminant analysis and completed as the first step to observe spectral features from both the negative and positive blood plasma samples to determine dataset groupings and score distributions, identify dominant peaks in the loadings, and detect outliers using the Hotelling’s T2 influence plot.

### 2.7 Soft independent modeling of class analogy analysis

Soft Independent Modeling of Class Analogy (SIMCA) is a supervised classification technique that builds a PCA model for each class in a calibration set. Test samples were then fitted to these models. By comparing the residuals to the maximum, test samples with allowed residuals are classified (Ghasemi et al., 2013). Blood plasma NIR spectra analysis was carried out using the SIMCA classification method. The impact of the difference between the positive and negative groups on spectral data was investigated. The spectra for positive and negative groups were revealed by SIMCA calibration. SIMCA classification for Paratuberculosis diagnosis based on ELISA test values was performed for the positive and negative groups.

### 2.8 Discriminant analysis

Discriminant analysis is a supervised and qualitative classification method that can classify new and unknown samples based on separate models for each group. It also helps to interpret differences between groups using LDA, unilinear discriminant analysis like QDA, or PLS-DA methods. LDA is a common technique considering both within-group and between-group variance. Decreasing the dimensionality of data increases the variance between and reduces the variance within classes, separating them. QDA applies when the variability of each group does not have the same structure (unequal covariance matrix), and the shape of the curve separating groups is not linear (Hastie et al., 2009).

LDA was used in the raw data and transformed spectra in the range of 1,300 nm to 1,600 nm and then separately for 12 water absorbance bands. Before applying LDA for classifying spectra into positive and negative JD classes, the dimensionality of each spectral dataset was reduced using PCA to overcome the constraint of requiring more objects (samples) and features (scores or PCs). PCs that captured more than 99% of the variance in the calibration dataset were selected for building the PCA-LDA model. LDA identifies similar spectral features for intra-class grouping and differential spectral features to separate dairy cattle’s healthy and infected blood plasma classes. It is reported that the PCA-LDA models from the confusion matrix evaluate the classification method as a percent (%) to describe the quality parameters of accuracy, sensitivity, and specificity.

The PCA-QDA method was then used to describe the nonlinear relationship between groups in raw data and transformed spectra in the range of 1,300 nm to 1,600 nm and 12 water absorbance bands separately.

PLS-DA analysis method was used to recognize the effect layers in discriminant models. All processes in the above were executed for this method. R^2^ and RMSC were used to determine the accuracy of the obtained PLS-DA models.

### 2.9 Support vector machine analysis

SVM is a supervised method that finds an optimal hyperplane or classifier and correctly separates objects into different classes as much as possible. SVM can effectively avoid over-fitting problems by leaving the most significant possible fraction of points from the same group on the same side and maximizing the distance of either group from the hyperplane and structural risk minimum mistake instead of the minimum error of the misclassification on the training set. Due to these advantages, SVM has gained extensive applications, including binary classification (Hastie et al., 2009).

### 2.10 Evaluation of classification methods

Quality parameters, such as accuracy, sensitivity, and specificity, were used to evaluate the classification method’s performance. The sensitivity test quantifies the model’s ability to correctly identify true positives of JD described by Equation 3, where TP represents true positive and FN represents false negative. A high sensitivity (>90%) is required when using the prediction model to identify severe but treatable diseases.

The model’s specificity shows its ability to identify healthy samples correctly. True negative was shown by Equation 4, where TN represents true negative and FP represents false positive. Also, the total accuracy is demonstrated by Equation 5.


(3) Sensitivity%=(TP/TP+FN)∗100

(4) Specificity%=(TN/TN+FP)∗100

(5) Total accuracy%=((TN∗(TN/TN+FP))+(TP∗(TP+FN∗100)))/TN+FP+TP+FN

### 2.11 Aquaphotomics

The contribution of water absorbance bands was investigated to detect the positive and negative blood plasma groups separately in JD. Therefore, a two-stage analysis is about all wavelengths of blood plasma samples in the range of 1,300 nm to 1,600 nm (first overtone of water), and the wavelength of only 12 water absorbance bands in this range was characterized as follows; C1, 1,336–1,348 (2ν_3_: H_2_O asymmetric stretching vibration); C2, 1,360–1,366 [OH−·(H_2_O)_1,2,4_: water solvation shell]; C3, 1,370–1,376 (ν_1_ + ν_3_: H_2_O symmetrical stretching vibration and H_2_O, asymmetric stretching vibration); C4, 1,380–1,388 [OH−·(H_2_O)_1,4_: water solvation shell, O_2_-·(H_2_O)_4_: Hydrated superoxide clusters, 2ν_1_: H_2_O symmetrical stretching vibration]; C5, 1,398–1,418 [water confined in a local field of ions (trapped water), S_0_: free water, water with free OH−]; C6, 1,421–1,430 (water hydration bond, H–OH bend and O–H … O); C7, 1,432–1,444 (S_1_: water molecules with 1 hydrogen bond]; C8, 1,448–1,454 [OH−·(H_2_O)_4,5_: water solvation shell]; C9, 1,458–1,468 (S_2_: water molecules with two hydrogen bonds, 2ν_2_ + ν_3_: H_2_O bending and asymmetrical stretching vibration); C10, 1,472–1,482 (S_3_: water molecules with three hydrogen bonds); C11, 1,482–1,495 (S_4_: water molecules with four hydrogen bonds); and C12, 1,506–1,516 (ν_1_: H_2_O symmetrical stretching vibration, ν_2_: H_2_O bending vibration, strongly bound water)3. Finally, the water bond study results are shown by aquagram using Equation 6 (Muncan and Tsenkova, 2019).


(6) $${\text{A}}^{\prime }\text{λ}=\left(\text{Aλ}-µ\text{λ}\right)/\text{σλ}$$

where A′λ is the normalized absorbance value displayed on the radar axis; Aλ is absorbance after scatter correction (multiplicative scatter correction using the mean of the dataset as a reference spectrum or standard normal variant transformation); µλ is the mean of all spectral; σλ is the standard deviation of all spectral; and λ are the selected wavelengths from Water Matrix Coordinates (WAMACs) regions corresponding to the activated water absorbance bands (Muncan and Tsenkova, 2019).

## 3 Results

### 3.1 Raw absorbance spectra of the positive and negative groups of blood plasma

Bovine blood plasma constitutes 55% of total blood volume (Mathew et al., 2023), with up to 92% water, 3% albumin and globulin, 4% immunoglobulin, 0.4% coagulants and fibrinogen, 0.5% minerals (sodium, potassium, bicarbonate, chloride, and calcium), and 0.07% of lipids related to hormone complex biofluid (Santos-Rivera et al., 2021).

**Figure 1:**
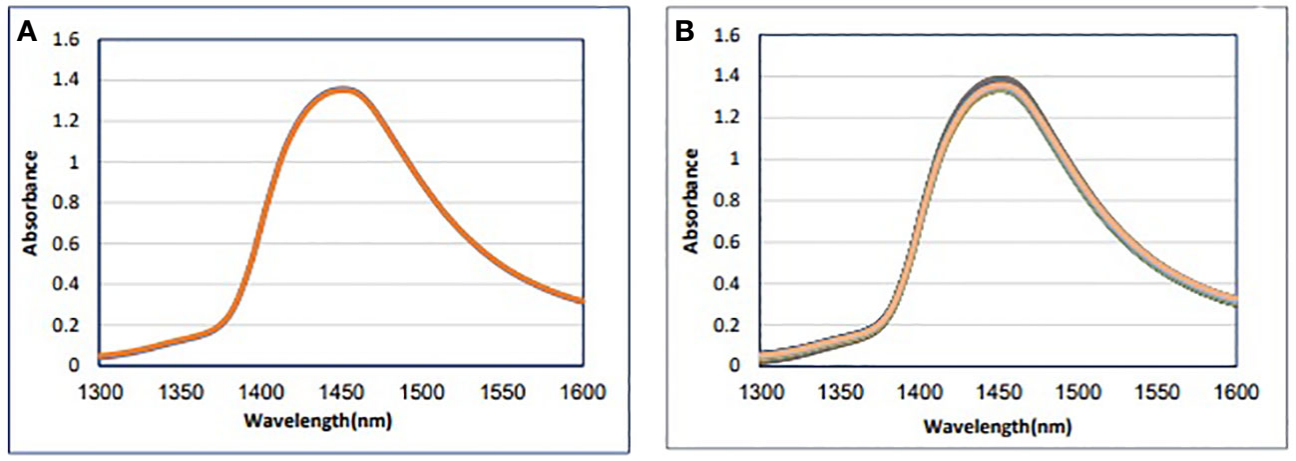
Absorbance NIR spectra dataset. **(A)** Raw and **(B)** mean of blood plasma (n = 79).

**Figure 1** shows blood plasma samples’ raw NIR absorbance spectra in the analyzed range of 1,300 nm to 1,600 nm. In this spectral region, these spectra appear identical, with the main feature being a dominant absorbance band around 1,450 nm attributed to the first overtone of OH stretching vibration (Vitalis et al., 2023). Because bovine blood plasma is 92% water, the spectra of blood plasma are similar to the water spectra (**Figure 1A**).

**Figure 2:**
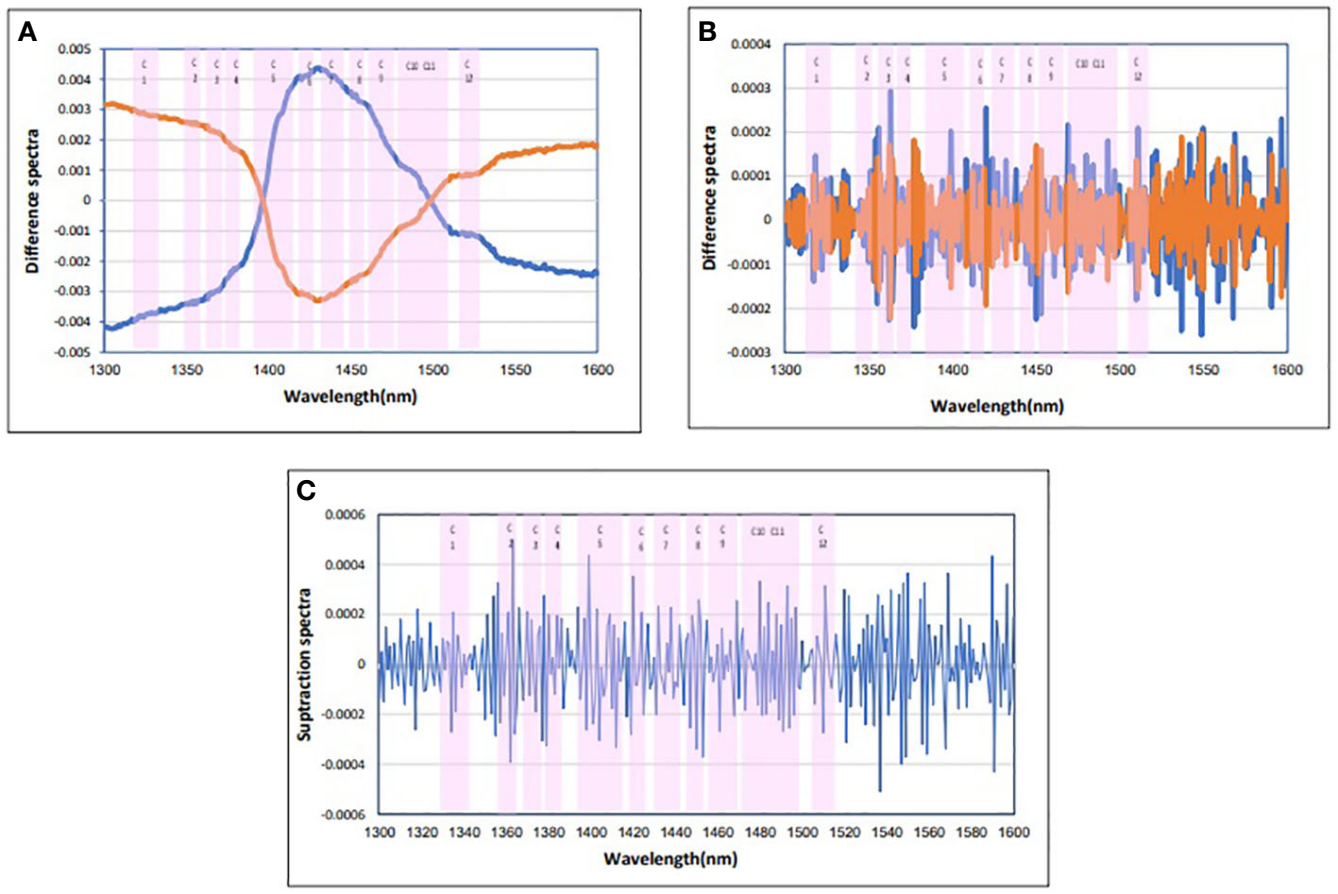
Subtraction spectra of the positive and negative groups of blood plasma. **(A)** Subtraction spectra of raw data of the positive and negative groups of blood plasma from the total mean spectra in Paratuberculosis. **(B)** Subtraction spectra of second derivative data from the positive and negative groups of blood plasma from the total mean spectra in Paratuberculosis. **(C)** Subtraction spectra of second derivative data of the positive (red) and negative (blue) groups of blood plasma from each other in Paratuberculosis. 12 water absorbance bands (pink).

Four calculations were usually performed in the initial data averaging and spectral subtraction evaluation. This was followed by loading in SIMCA and regression coefficient in PLS-DA analysis to enhance the subtle changes at specific water absorbance bonds in the spectra of blood plasma samples (Tsenkova et al., 2018). The raw data average of blood plasma for each group is not interested (**Figure 1B**). To emphasize the subtle differences, the raw data average spectra in **Figure 2A**, and the positive and negative groups’ second derivation were subtracted from the total average of all spectra (**Figure 2B**). Another way to enhance the differences is to calculate the difference spectrum between the average spectra of positive and negative groups’ second derivation (**Figure 2C**). This spectral subtraction enhanced the differences between the two groups in raw data, and the most considerable difference is in the regions around 1,300 nm to 1,388 nm, 1,421 nm to 1,495 nm, and 1,506 nm to 1,600 nm. In the second derivation spectral subtraction, all 12 water bonds are active, and the differences are noticeable.

### 3.2 PCA: exploratory analysis of Johne’s disease effects on spectra of blood plasma

**Figure 3:**
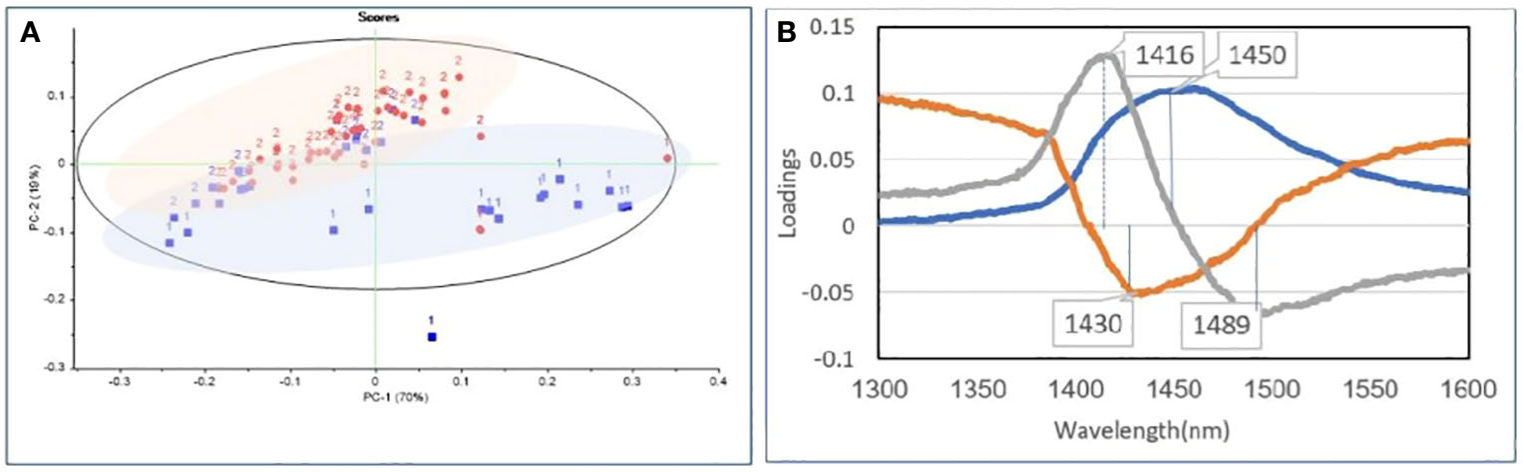
Principal component analysis of blood plasma. **(A)** PCA of raw data of blood plasma NIR spectra (1,300 nm to 1,600 nm) from the positive (red) and negative (blue) groups in Paratuberculosis farm1 (1) and farm2 (2). **(B)** PCA loading plot for baseline pretreatment samples from the positive and negative groups containing the first three PCs explaining 100% of the variance. PC1 (blue), PC2 (red), and PC3 (gray).

The PCA is an unsupervised MVA that can reduce the dimensionality of datasets, explain variation in the data by ignoring data labels, detect patterns in spectral behavior, and find excluded data. PCA results were presented as scores and loadings plots. **Figure 3** depicts the similarities and differences in chemical complexes containing OH, CH, and NH bonds interacting with NIR light in transformed spectra of bovine blood plasma in the range of 1,300 nm to 1,600 nm in the PCA score plot. The PCA loadings revealed the dominant peaks influencing the trends in the score plot (**Figure 3**), which were related to the OH in water bonds. According to the PCA analysis of the raw data, two herds were separated entirely, and the positive and negative groups were separated as expected from unsupervised analysis, so the positive and negative classes were placed above and below the graph, respectively.

PC1 represented the most variation in the data (70%) among the first three PC loadings, with PC2 and PC3 representing the second and third most variation (19% and 10%, respectively). Two outliers were discovered in Hotelling’s T2. Furthermore, these spectra were removed before averaging and spectral subtraction. Next, by baseline pretreatment, the first three PC loadings, PC1 (89%), PC2 (10%), and PC3 (1%), explained 100% of the variance.

### 3.3 SIMCA analysis for detection of cattle response to Johne’s disease: discrimination of positive and negative blood plasma

**Figure 4:**
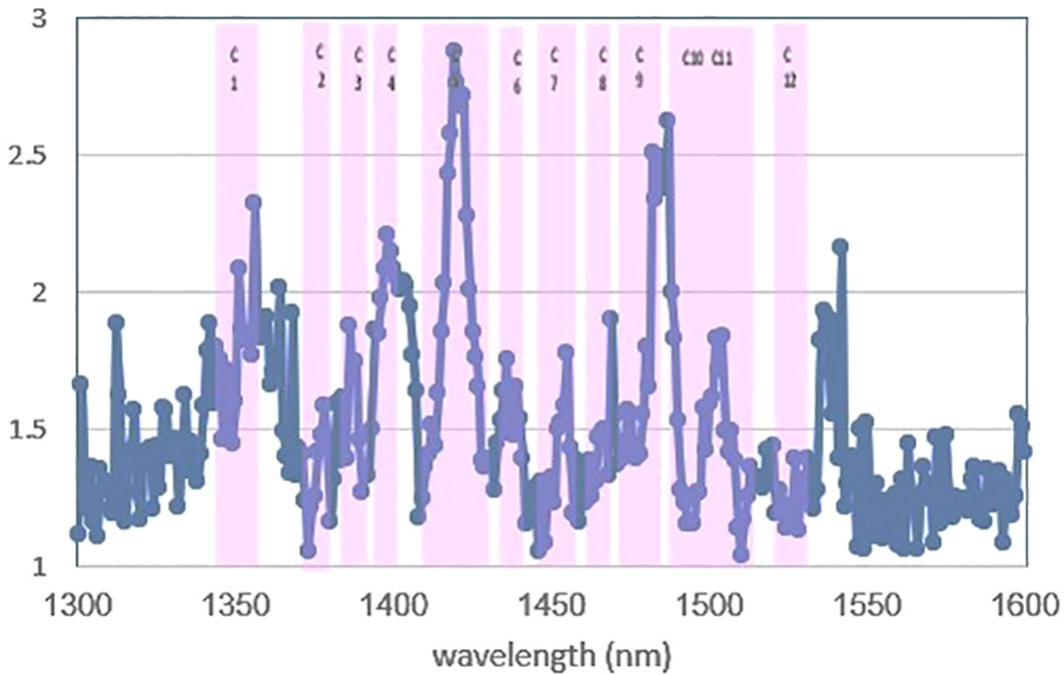
Discriminating powers of SIMCA analysis of data with normalized pre-treatment. All of the 12 water absorbance bands are active in discriminating of models.

First, SIMCA was applied to perform a supervised classification of blood plasma spectra according to the negative and positive classes in the range of 1,300 nm to 1,600 nm. In raw data, the test set’s classification accuracy, sensitivity, and specificity were 83.5%, 73%, and 100%, respectively. Total accuracy for SNV, MSC, baseline, and spectroscopic preprocessing was 89%, sensitivity was 82%, and specificity was 100%. Finally, the data preprocessing normalization results were the best, with the data having 94.5% total accuracy, 91% sensitivity, and 100% specificity by the first three PCs for the two groups (**Table 2**). Discriminating powers of SIMCA data analysis with normalized pretreatment show that the wavelengths contributed to the successful separation of blood plasma at positive and negative groups and that all wavelengths are active in discriminating models in **Figure 4**.

The absorbance value at the WAMACs (12 water absorbance bands) was used to calculate the SIMCA model of blood plasma spectra. The total accuracy in raw data was 89%, with the sensitivity and specificity of 82% and 100%, respectively. Baseline pretreatment resulted in a total accuracy of 94.4%, sensitivity of 100%, and specificity of 85.7%, whereas smoothing pretreatment resulted in a total accuracy of 94.5%, sensitivity of 91%, specificity of 100% (**Table 2**).

### 3.4 LDA analysis for detecting dairy cattle response to Johne’s disease of blood plasma

LDA is the simplest classification method that supervises dimensionality reduction techniques, classifies data simultaneously, and focuses on finding a feature subspace that maximizes group separability. The variables with binary or multiclass labels are the target of LDA (Ghasemi et al., 2013).

The PCA–LDA was conducted on spectra of blood plasma from both the positive and negative groups simultaneously. Three PCs were chosen from a total of 10 PCs, explaining 100% of the variance in the PCA of the calibration sets for creating the discriminant and prediction equations in full data, calibration for internal validation, and testing for external validation.

In internal validation, the specificity and sensitivity of LDA were 87.5% and 91% for full raw data and were both 88% for calibration data, respectively. The positive and negative groups separated with 100% total accuracy, sensitivity, and specificity when using the prediction model for external validation. In the range of 12 water bonds, full data and calibration had 93.5% and 91.5% total accuracy, 97% and 96% specificity, and 91% and 88% sensitivity, respectively. External validation revealed that raw data had 100% total accuracy, sensitivity, and specificity.

Increased accuracy, sensitivity, and specificity in 12 water absorbance bands range analysis with the range of 1,300 nm to 1,600 nm in internal validation and similarity in external validation results demonstrated the importance of water in detecting healthy and infected groups (**Table 2**).

### 3.5 QDA analysis for detection of dairy cattle response to Johne’s disease of blood plasma

Another type of discriminant analysis is QDA. When each group’s variability does not have the same structure (unequal covariance matrix) and the curve shape separating groups is not linear, QDA provides a better classification model.

In internal validation, QDA in the range of 1,300 nm to 1,600 nm for the total wavelength of raw data in the negative and positive groups was 100% and 98% and that for calibration data was 100% and 97%, respectively, for specificity and sensitivity. External validation separates the positive and negative groups based on 100% total accuracy, sensitivity, and specificity.

In internal validation, QDA in WAMACs in blood plasma has 97% and 98% sensitivity and 100% and 100% specificity for full data and calibration, respectively. External validation using SNV, MSC, Baseline, smoothed, and normalized pretreatment yielded 100% total accuracy, sensitivity, and specificity in Paratuberculosis detection (**Table 2**).

### 3.6 PLS-DA analysis for detection of dairy cattle response to Johne’s disease of blood plasma

PLS-DA, as a supervised method in discriminant analysis, is a type of qualitative calibration in which the category group variable is used for classification rather than continuous measurement, as in quantitative calibration, using partial least squares regression methods.

Data were systematically divided into internal validation (a full dataset and calibration set) and external validation (a prediction set). **Table 1** shows the number of samples and method characteristics in the full data, calibration, and prediction sets. The relationship between the actual and predicted class was analyzed by PLS-DA in the 1,300 nm to 1,600 nm region using absorbance values of blood plasma spectra, according to the results of the ELISA test as reference values. Internal validation using normalized preprocessing yielded 100% specificity and 98% sensitivity for full data and 100% specificity and 97% sensitivity for calibration. R^2^ values were 78% and 84%, respectively.

**Figure 5:**
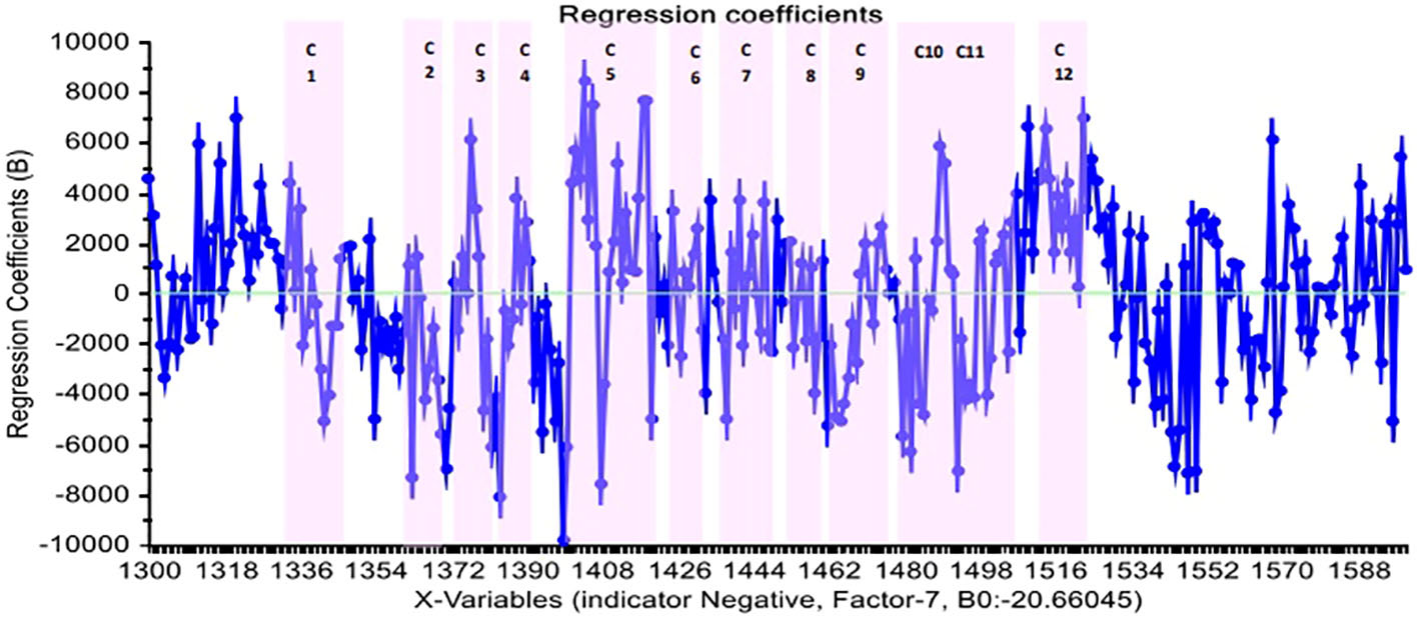
The regression coefficient of wavelengths in the PLS-DA method and normalized pretreatment for distinct positive and negative groups of blood plasma in Paratuberculosis.

**Figure 5** depicts the regression coefficient of the wavelength variables using the PLS-DA method and normalized preprocessing with seven PCs. In this diagram, each variable with a high regression coefficient was included in the model, and the highest regression coefficients relate to the water absorbance bands.

Internal validation of PLS-DA in 12 water absorbance bands of 1,300 nm to 1,600 nm region using absorbance values at the WAMACS of blood plasma spectra achieved 94% and 91% sensitivity and specificity for full data and 96% and 94% sensitivity and specificity for calibration data, respectively. These figures were 100% and 82% in external validation, respectively. When these results are compared to the range of 1,300 nm to 1,600 nm, all the specificity and 82% of the sensitivity belong to the water absorbance bands and can be recognized by them (**Table 2**).

### 3.7 SVM analysis for detection of cattle response to Johne’s disease of blood plasma

SVM has been used to detect Paratuberculosis and health groups as a powerful supervised method. According to SVM analysis, the best prediction equation obtained from the first derivative model, which was the result of internal validation of full data and calibration, contained 97% and 96% specificity with 100% sensitivity, and the total accuracy, sensitivity, and specificity for external validation were 100%. In the separation of negative and positive groups, these models yielded a high coefficient of determination (R²) of 96% and a low root mean square error of prediction (RMSEP) of 0.1% (**Table 2**).

This time, SVM was performed on the basis of aquaphotomics. In comparison to the data obtained from SVM by 12 water absorbance bands (WAMACs) with the results obtained from all wavelengths in the range of 1,300 nm to 1,600 nm, in internal validation models with normalized pretreatment, full data and calibration have 98% and 97% sensitivity and 100% specificity. The coefficient of determination, R² = 96%, and RMSEP = 0.1 were obtained in separating the negative and positive groups. External validation shows that, in the model with normalized pretreatment, total accuracy in separating positive and negative groups increases (to 100%).

External validation shows that the model with normalized pretreatment achieved an increased total accuracy (100%) in separating positive and negative groups for Paratuberculosis, demonstrating the high contribution of water bonds in the results of the SVM models in the range of 1,300 nm to 1,600 nm.

These findings suggested that biochemical changes related to antibody amount in blood plasma as a result of the dairy cattle response to MAP infection in JD could be accurately detected and classified using NIRS and aquaphotomics (**Table 2**).

### 3.8 Aquagrams

Finally, the difference in the water structure in the blood plasma of the negative and positive groups was determined. It can be investigated using the aquagram of the active water absorbance bands created during the disturbance, which is JD in this case. The aquagram displays normalized absorbance values from MSC pretreatment at water absorbance bands on the axes originating from the center of the graph to identify the water absorbance bands that responded strongly to JD. WAMACS absorbance values were used for axes. By comparing the aquagrams for the positive and negative groups, the relationship between two groups of infected and healthy individuals with WASP was estimated.

**Figure 6:**
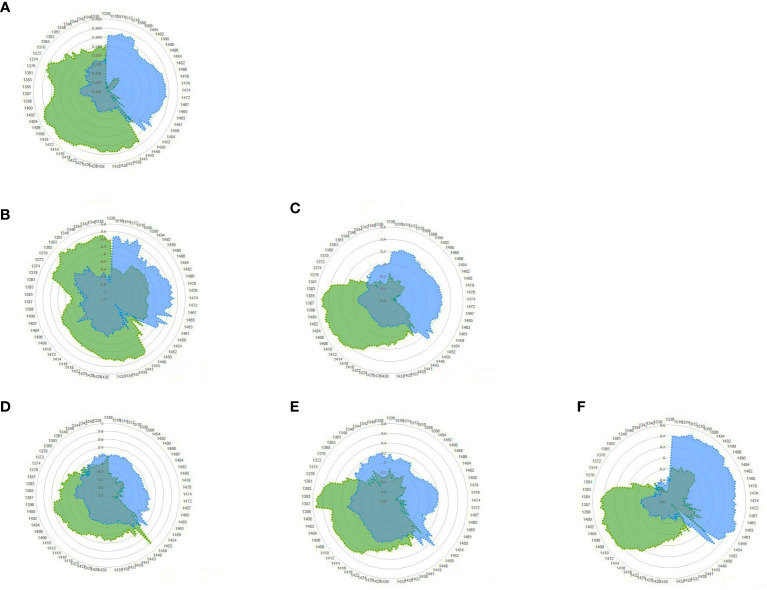
Aquagram of blood plasma dairy cattle with MSC pretreatment for Paratuberculosis, showing evidence different in WASPs of the positive (blue) and negative (green) groups. **(A)** Total; **(B)** Herd1; **(C)** Herd2; **(D)** Herd2-Week1; **(E)** Herd2-Week2; **(F)** Herd2-Week3.

Based on the results of mean spectrum difference, SIMCA, and PLS-DA, all WAMACS wavelengths distinguish between the positive and negative groups and are thus used in drawing the aquagram. According to the diagram, active water absorbance bands in the negative class primarily include C1: water with asymmetric stretching vibration, C2: water solvent shell, and C3: water bonds with symmetric and asymmetric stretching vibration, C4: hydrated superoxide clusters and symmetrical stretching vibration, C5: containing free water, C6: have free OH bonds and hydrated water, and C7: bonds with a hydrogen bond were increased. However, in the positive class, these water absorbance bands are significantly reduced and water absorbance bands C8: water solvation shell, C9: water molecules with two hydrogen bonds, C10: with three hydrogen bonds, C11: with four hydrogen bonds, and C12: with stronger bonds Increased. In other words, the effect of changes in antibody blood plasma causing JD can be observed in water absorbance bands spectral patterns (WASPs), where the free water proportion, hydrated, and having a hydrogen bond in the blood plasma is significantly reduced. Also, the water molecules are organized in groups of 3 or 4 with the formation of more and stronger hydrogen bonds. Furthermore, as the amount of antibodies in JD changed, the proportion of water molecules with symmetric and asymmetric stretching vibrations decreased (**Figure 6**).

## 4 Discussion

JD changes the amounts of antibodies in dairy cattle’s blood plasma. An ELISA kit can detect these changes. Due to the characteristics of JD and the lack of 100% diagnostic accuracy of this kit, animals with a history of JD were tested for three consecutive weeks. However, animals with three similar test results and an index of less than 20 were considered healthy or negative. Animals with an index greater than 100 were classified as infected or positive. The two groups were chosen on the basis of the results of three consecutive weekly ELISA blood plasma tests in dairy cows: a healthy group with an index of ≤20 and an infected group with an index of ≥100. The NIR spectrum profile was used to develop five classification models based on the antibody index in plasma and the biochemical changes caused by the disease in the blood plasma, particularly in the structure of the water molecules that make up the plasma. These models diagnosed Paratuberculosis and distinguished healthy and infected samples with 100% total accuracy, sensitivity, and specificity. Finally, the aquagram was obtained, which shows the changes in the 12 water absorbance bands in healthy and infected dairy cattle.

The first, for the positive and negative groups, separate datasets were created. The null hypothesis (no biological signature can distinguish samples from these two groups) was tested by NIRS and aquaphotomics approach to the unsupervised and supervised analysis in two ranges: 1,300 nm to 1,600 nm and only in 12 water absorbance bands in this range.

NIRS can provide information on the chemical and physical composition of raw materials such as milk, particularly on portable instruments that can be used directly on the dairy farm (Evangelista et al., 2021). Additionally, NIRS is used to detect genetically modified organisms. Aquaphotomics is a novel field that could be explored for monitoring them (Sohn et al., 2021).

The physiological and metabolic changes are the foundation for NIRS profiling to distinguish between healthy and infected animals. It can be used to detect and monitor infection in dairy cattle. The profile of NIR spectra reflects the animal’s immune response to JD agent by applying MVA and the aquaphotomics approach based on the biochemical changes that caused the difference between the blood plasma of healthy and infected animal (Meilina et al., 2009; Kinoshita et al., 2012b; Ramirez-Morales et al., 2021). In 2012, *Mycobacterium tuberculosis* was detected by infrared and near-infrared spectroscopy (Pesala et al., 2012).

This study analyzed the blood plasma spectra using PCA, LDA, QDA, PLS-DA, and SVM methods by ELISA test as a reference test for calibration. This analysis reveals information about biochemical imbalances caused by diseases that affect the composition of blood plasma. This effect causes the changes in the structure of the water in the blood plasma, characterized by the reuse of PCA, LDA, QDA, PLS-DA, and SVM methods in the range of 12 bonds of water and can be seen in the aquagram.

The raw data spectrum and the average of the negative and positive groups followed the water spectral pattern, with a peak at 1,450 nm, confirming the high proportion of water in blood plasma compounds.

According to the PCA analysis in raw data, the two herds were separated entirely, and the positive and negative groups were separated as expected from unsupervised analysis. Accordingly, the positive and negative classes were at the top and bottom of the graph, respectively. By baseline pretreatment, the first three PC loadings, PC1, PC2, and PC3, explained 89%, 10%, and 1% of the dataset variance, respectively (100%).

The SIMCA method was studied for blood plasma in the range of 1,300 nm to 1,600 nm using raw data and some pretreatments, and the normalized preprocessing produced the best results: 94.5% total accuracy, 91% sensitivity, and 100% specificity by the first three PCs for two groups.

The SIMCA models calculated using the absorbance value at the WAMACs (12 water absorbance bands) and smoothing pretreatment data had a 94.5% total accuracy, a 91% sensitivity, and a 100% specificity. The similarity of the results in the range of 1,300 nm to 1,600 nm and in 12 water absorbance bands demonstrates the effective and decisive role of water bonds changes in distinguishing healthy and infected groups in NIRS.

The PCA and SIMCA models were used to diagnose mastitis in dairy cattle (Tsenkova and Atanassova, 2001; Kinoshita et al., 2012b; Ramirez-Morales et al., 2021) by identifying NIR features of blood plasma.

Internal validation of the LDA model in full raw data and calibration yielded 89.5% and 88% total accuracy, 87.5% and 88% specificity, and 91% and 82% sensitivity, respectively. External validation of positive and negative groups was separated with 100% total accuracy, sensitivity, and specificity using the prediction model. Full data and calibration had 93.5% and 91.5% total accuracy, 97% and 96% specificity, and 91% and 88% sensitivity, respectively, in the 12 water absorbance bands. However, the raw data had 100% total accuracy, sensitivity, and specificity in external validation. Increased accuracy and specificity in 12 water absorbance bands analysis compared to the range of 1,300 nm to 1,600 nm in internal validation and similarity in sensitivity and external validation results demonstrate the importance of water in detecting healthy and infected groups.

In the QDA model, the results of full data and 12 water absorbance bands were the same in internal and external validation. In the internal evaluation, the raw data in the full range and the preprocessed data with MSC achieved 99% and 98% total accuracy, 98% and 97% sensitivity, and 100% specificity, respectively. Also, the total accuracy, sensitivity, and specificity were 100% in the external validation, demonstrating the decisive role of water in separating the two groups.

In 2021, a study with 18 PCs used NIRS and LDA with aquaphotomics to diagnose Pneumonia in dairy calves with 98.5% total accuracy, 97.5% sensitivity, and 99.6% specificity (Santos-Rivera et al., 2021). Another study in 2023 used NIRS, LDA-PCA, PLS regression, and aquaphotomics to monitor lettuce’s freshness during cold storage. This study found that WAMACs reflected all information about the freshness of lettuce during storage. This study also showed that WASPs can be used as a multidimensional biomarker to monitor changes during storage (Vitalis et al., 2023).

In the 1,300 nm to 1,600 nm region, PLS-DA in internal validation by normalized preprocessing for full data achieved 100% specificity and 98% sensitivity, and with the calibration, 100% specificity and 97% sensitivity. The R^2^ was 78% and 84%, respectively. Using absorbance values at the WAMACS of blood plasma spectra, in internal validation, sensitivity and specificity were achieved at 94% and 91% for full data and at 96% and 94% for calibration data, respectively. These amounts, in external validation, were 100% and 82%, respectively. By comparing these results with the range of 1,300 nm to 1,600 nm, it is clear that all the specificity and 82% of the sensitivity belong to the water bonds and it can be recognized by them.

NIRS and aquaphotomics used the PLS model to detect estrus in dairy cattle (Takemura et al., 2015; Iweka et al., 2020). The water absorbance bands in panda and orangutan urine spectrum were used as a biomarker in estrus detection. Based on the PLS-PCA method, this study showed that the female panda’s small estrous hormone concentration was measured and detected using water absorbance bands in the range of 1,300 nm to 1,600 nm, and the aquagram showed these changes (Kinoshita et al., 2012b, 2015). Using first derivative pretreatment and PLS-PCA method with cross-validation models, another study has managed to detect estrous cycle stages by horse blood serum through NIRS and aquaphotomics (Agcanas et al., 2017; Vance et al., 2017).

The best prediction equation was obtained from the first derivative model for the SVM analysis. This model provided 97% and 96% specificity with 100% sensitivity in the internal validation of full data and calibration. The external validation resulted in a total accuracy, sensitivity, and specificity of 100%. In separating negative and positive groups, these models have a high coefficient of determination (R^2^) of 96% and a low error coefficient (RMSEP) of 0.1%. Following that, the SVM models were used in internal validation models in water absorbance bands (WAMACs) with normalized pretreatment full data and calibration with 98% and 97% sensitivity and 100% specificity with R^2 =^ 96%, RMSEP = 0.1 in the separation of negative and positive groups. External validation shows that the model with normalized pretreatment increases total accuracy, with 100% obtained in separating the positive and negative groups for Paratuberculosis. This result indicates a high contribution of water bonds in the results of the SVM models in the range of 1,300 nm to 1,600 nm.

Early diagnosis of type 2 diabetes based on NIRS and SVM model with aquaphotomics approach showed 97.22% accuracy, and the specificity and sensitivity were 95.65% and 100%, respectively, by the first derivative pretreatment. This study demonstrates that combining NIRS with aquaphotomics is effective for developing an accurate and rapid early diabetes diagnosis model (Li et al., 2020).

NIRS was used in 2006 as a new approach to diagnose Paratuberculosis in dairy cattle. This study used NIRS and the artificial neural network method, with FC and serum test ELISA as a reference test. This research used NIRS as an indirect test for Paratuberculosis infection in cattle. However, it was unclear which parameters or substances in the serum that the NIRS discriminates (Norby et al., 2006).

The total accuracy of using NIRS to diagnose JD in dairy cattle with a 95% confidence margin is 99%, 98%, and 100% in full data, calibration, and test, respectively. The method’s sensitivity and specificity were 100%, which is comparable to the sensitivity of current diagnostic models, such as ELISA test (7%–94%), FC (20%–74%), and PCR (4%–100%). The best model uses blood plasma spectra from QDA in the range of 1,300 nm to 1,600 nm or only WAMACS.

**Figure 7:**
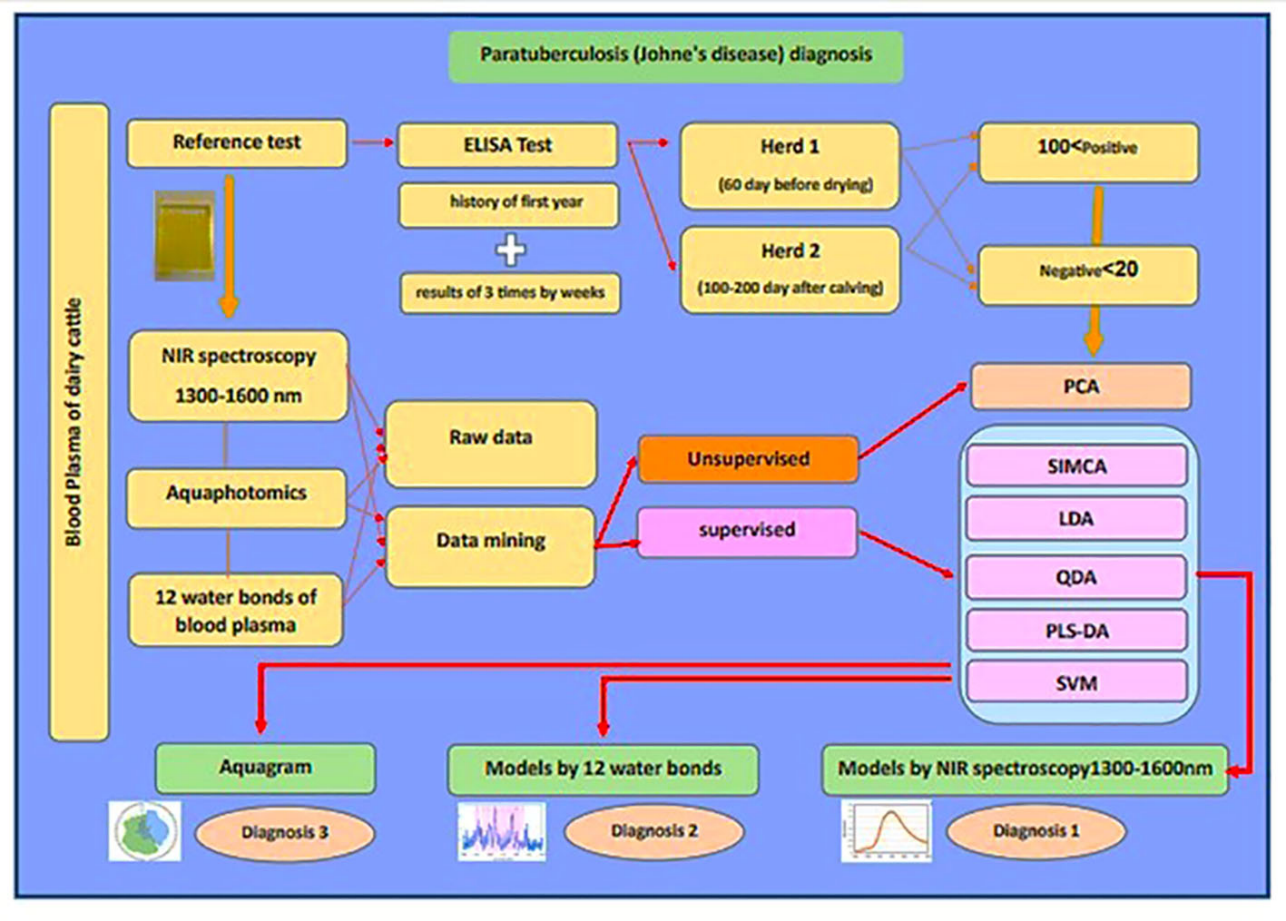
The brief of research “A novel diagnostic approach to Paratuberculosis in dairy cattle using near-infrared spectroscopy and aquaphotomics”.

The accuracy and quality of diagnosis utilizing advanced diagnostic facilities such as aquaphotomics and advanced diagnostic equipment such as NIRS improves. These facilities assist farmers in detecting JD in the early stages and providing appropriate treatment. **Figure 7** shows the brief of this study.

## 5 Conclusion

In this study, for the first time, the two groups were chosen on the basis of the results of three consecutive weekly ELISA blood plasma tests in dairy cows: a healthy group with an index of ≤20 and an infected group with an index of ≥100. The NIR spectrum profile was used to develop five classification models based on the antibody index in plasma and the biochemical changes caused by the disease in the blood plasma, particularly in the structure of the water molecules that make up the plasma. These models diagnosed Paratuberculosis and distinguished healthy and infected samples with 100% total accuracy, sensitivity, and specificity. Finally, the aquagram was obtained, which shows the changes in the 12 water absorbance bands in healthy and infected dairy cattle, as a multidimensional biomarker. Our findings show that NIRS and aquaphotomics approach can lead to non-invasive, fast, and accurate diagnosis of Paratuberculosis in dairy cattle.

In this study, we found that NIRS by supervised methods can be accurate to diagnosis JD. Completely, we found that aquaphotomics by using only 12 water absorbance bands of blood plasma can detected the healthy and infected dairy cattle. In the last, aquagrams show that WASPs can be used as a multidimensional biomarker to monitor changes of antibody in blood plasma and the very simple, fast, and accurate way to detection of healthy and infected dairy cattle by Paratuberculosis. According to the finding of this study, we introduced the novel methods to diagnosis of Paratuberculosis in dairy cattle by means of blood plasma with NIRS and aquaphotomics. Our long-term goal is to create a diagnostic strategy for the detection of dairy cattle in the four stages of JD by this non-invasive, accurate, and fast diagnostic method *in vivo*, thereby contributing to the sustainability of the genetic resources and the global health.

## Data availability statement

The original contributions presented in the study are included in the article/supplementary material, further inquiries can be directed to the corresponding author/s.

## Ethics statement

This research approved by the SRBIAU- Institution Animal Science (SRB -11-3997).

## Author contributions

SB: Conceptualization, Data curation, Formal analysis, Investigation, Methodology, Resources, Software, Validation, Visualization, Writing – original draft. AP: Conceptualization, Data curation, Investigation, Methodology, Project administration, Supervision, Validation, Writing – review & editing. RM: Conceptualization, Data curation, Investigation, Methodology, Project administration, Supervision, Validation, Writing – review & editing.
